# Hispanic/Latino(a) Aging With HIV: Insights From The MWCCS Cohort

**DOI:** 10.1093/geroni/igaf122.4130

**Published:** 2025-12-31

**Authors:** Mirza Ishrat Noor, Mark Kuniholm, Allison Appleton, Jessica Islam, Marlene Camacho-Rivera, Gypsyamber D’Souza, Deborah Gustafson, Elizabeth Vásquez

**Affiliations:** University at Albany (SUNY), Albany, New York, United States; University at Albany (SUNY), Albany, New York, United States; University at Albany (SUNY), Albany, New York, United States; H. Lee Moffitt Cancer Center and Research Institute, Tampa, Florida, United States; SUNY Downstate Health Sciences University, Brooklyn, New York, United States; Johns Hopkins University, Baltimore, Maryland, United States; SUNY Downstate Health Sciences University, Brooklyn, New York, United States; University at Albany (SUNY), Albany, New York, United States

## Abstract

Hispanic/Latino (H/L) individuals living with HIV are an understudied and diverse population with significant health disparities compared to non-H/L individuals. We examined frailty and cardiovascular disease (CVD) risk among H/L and non-H/L White adults aged ≥40 years in the MACS/WIHS Combined Cohort Study (MWCCS). The sample included 621 H/L and 1,293 non-H/L White participants. Frailty was defined using the Fried Frailty Phenotype (FFP), with a score ≥3/5 indicating frailty. Ten-year CVD risk was categorized as low (< 5%), borderline (5–7.4%), intermediate (7.5–19.9%), or high (≥20%). Adjusted odds ratios (aOR) and 95% confidence intervals (CI) assessed associations between ethnicity and outcomes, with models stratified by sex and adjusted for HIV status, age, education, BMI, depression, diabetes, and hypertension. H/L participants were more likely to be women (53.6% vs. 15.9%), frail (22.5% vs. 11.8%), and living with HIV (73.3% vs. 50.7%), but less likely to have hypertension (46.9% vs. 66.3%) or high CVD risk (7.5% vs. 18.4%). Among men, H/L participants had significantly lower odds of intermediate (aOR: 0.32, CI: 0.18–0.57) and high (aOR: 0.20, CI: 0.09–0.45) CVD risk but higher odds of frailty (aOR: 3.54, CI: 1.65–7.58) than non-H/L Whites. Among women, no significant differences were observed in frailty or CVD risk by ethnicity. Despite lower CVD risk, H/L men had higher frailty, suggesting potential sex-specific disparities that require further investigation.

